# Supplementary material for: Differences in Internet Use and eHealth Needs of Adolescent and Young Adult Versus Older Cancer Patients; Results from the PROFILES Registry
Source: Cancers (Basel). 2021 Dec 16;13(24):6308. doi: 10.3390/cancers13246308 (PMC8699784; doi:10.3390/cancers13246308)

Table S1. Characteristics of the participants of AYAs (n=299) and older adults (n=270) stratified by POP and OCC groups.

		Adolescents and young adults (18-39 years)				Older adults (40 + of age)				
		Population based (POP) (n=198)		Online cancer community (OCC) (n=101)		Population based (POP) (n=142)		Online cancer community (OCC) (n=128)		
		N	%	N	%	N	%	N	%	Significant different
Gender	Female	143	72	91	90	128	90	93	73	a b c d
	Male	55	28	10	10	14	10	35	27	
Age (at diagnosis), (mean (sd))		32.1 (5.8)		31.1 (5.5)		57.4 (9.9)		53.9 (7.9)		b c d
	18-24 years	30	15	15	15	0	0	0	0	
	25-39 years	53	27	43	43	0	0	0	0	
	40-64 years	0	0	0	0	109	77	112	88	
	65 + years	0	0	0	0	33	23	16	13	
Years since diagnosis (mean ± SD)		7.6 (1.7)		3.0 (2.4)		2.8 (0.6)		3.4 (1.9)		a b c
	0-2 year(s)	1	1	63	62	124	87	68	53	
	>2-5 years	55	28	25	25	18	13	53	41	
	>5 years	73	37	13	13	0	0	7	5	
Education level	Primary school	0	0	0	0	3	2	0	0	b c d
	Secondary school	71	36	37	37	104	74	65	51	
	College/university	126	64	64	63	34	24	62	49	
Marital status (at time of questionnaire)	Partner	156	79	77	76	119	84	101	80	
Type of cancer	Brain cancer	12	6	4	4	0	0	0	0	
	Breast cancer	66	33	39	39	60	42	51	40	
	Bone cancer	4	2	0	0	0	0	0	0	
	Gastrointestinal cancer	8	4	5	5	0	0	21	16	b c
	Gynacological cancer	28	14	15	15	54	38	14	11	b c
	Head and neck cancer	2	1	0	0	0	0	0	0	
	Leukemia	12	6	3	3	0	0	0	0	
	Lung cancer	1	1	2	2	0	0	12	9	
	Lymphoma	27	14	19	19	28	20	16	13	
	Sarcoma	4	2	4	4	0	0	0	0	

	Skin cancer	6	3	3	3	0	0	7	5	b c
	Testicular cancer	20	10	0	0	0	0	0	0	a
	Thyroid cancer	9	5	3	3	0	0	0	0	
	Urological cancer	1	1	3	3	0	0	0	0	
	Other[1]	4	2	3	3	0	0	7	5	b
Treatment modality	Surgery	138	70	77	76	106	75	83	65	
	Chemotherapy	137	69	74	73	76	54	92	72	b c
	Radiotherapy	112	57	52	51	59	42	72	56	b c
	Hormone therapy	33	17	23	23	30	21	38	30	
	Immunotherapy	17	9	16	16	0	0	27	21	b c
	Stem cell transplantation	10	5	5	5	0	0	2	2	c
	Targeted therapy	4	2	4	4	29	20	5	4	b c
	Other[2]	5	3	2	2	0	0	4	3	b

[1] Neuroendocrine tumor, mesothelioma, trophoblast tumor, multiple myeloma, esthesioneuroblastoma and thymus cancer.

[2] Radio active iodine therapy and no therapy or active surveillance

NA=not applicable

[a] significant different between AYAs (POP vs OCC)
[b] significant different between older adults (POP vs OCC)
[c] significant different between POP-groups (AYAs vs older adults)
[d] significant different between OCC-groups (AYAs vs older adults)

Figure S1A-D. Search frequency for information about cancer on the internet during different phases of disease according to AYAs and older adults stratified by POP and OCC groups.

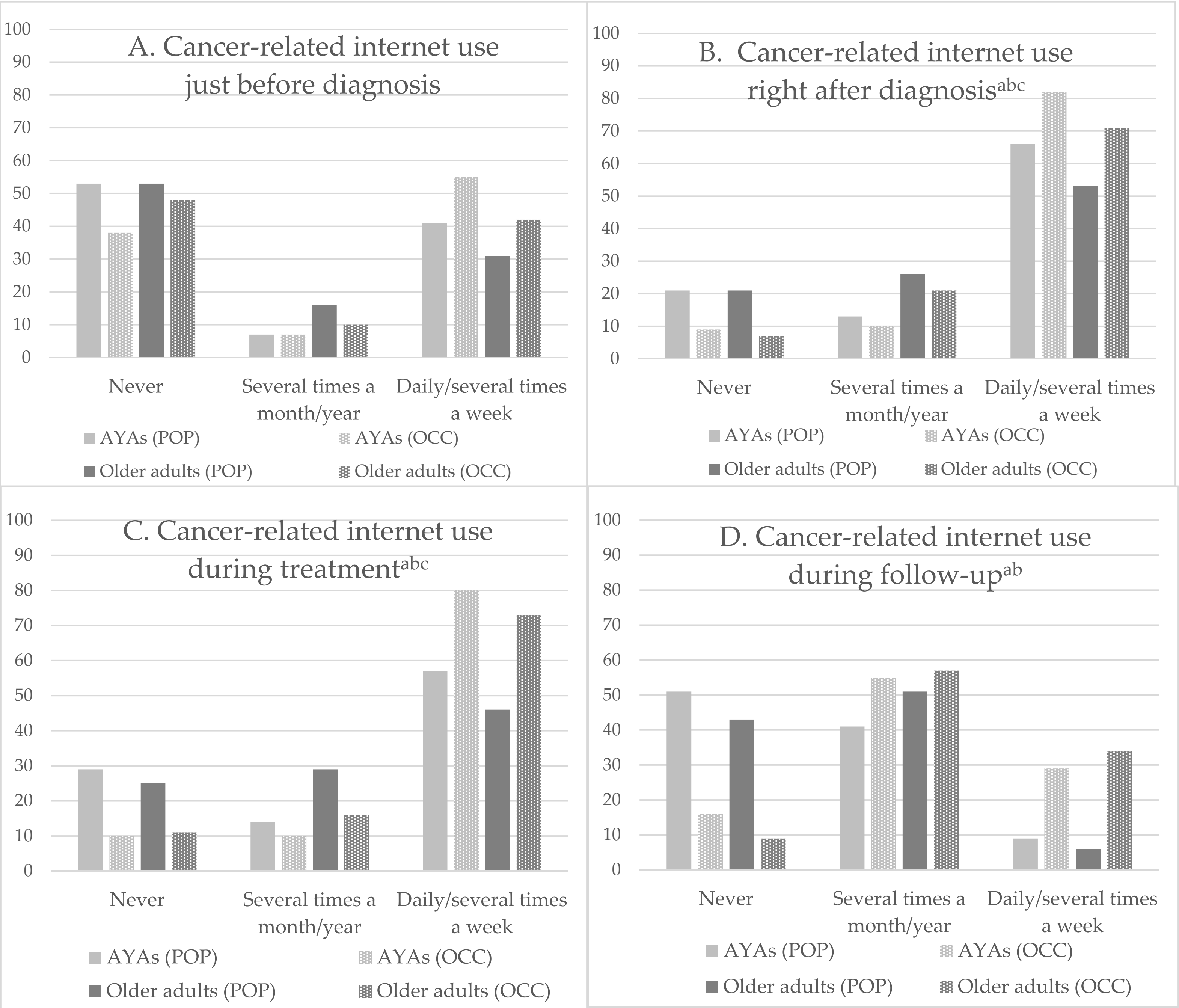

Table S2. Medical and psychosocial topics searched for on internet during and after treatment by AYAs and older adults stratified by POP and OCC groups.

	**Adolescents and young adults (18-39 years) n=299**				**Older adults (40 + of age) n=270**				
	**Population based (POP) (n=198)**		**Online cancer community (OCC) (n=101)**		**Population based (POP) (n=142)**		**Online cancer community (OCC) (n=128)**		Significant different
**Medical topics**	N	%	N	%	N	%	N	%	
Type of cancer	119	62	65	68	106	80	115	92	b c d
Treatments	145	75	80	84	89	69	114	90	b
Consequences of treatment in general	143	74	85	89	92	73	109	86	a b
Alternative or complementary therapies	62	32	30	32	16	13	37	30	b c
Finding a doctor	44	23	28	29	13	10	37	30	b c
Finding a hospital	58	30	34	36	21	17	46	37	b c
Trials and/or research	48	25	37	39	27	22	66	53	a b d
Treatment guidelines	111	58	77	81	59	46	81	65	a b d
Cancer genetics and heritability	107	55	66	69	62	50	72	57	a b d
Fertility	83	43	53	56	1	1	5	4	a b c d
End of life	43	22	31	33	6	5	30	25	b c
**Psychosocial topics**	N	%	N	%	N	%	N	%	
What can I do myself	121	63	74	80	63	50	87	73	a b c
Sexuality and intimacy	88	46	59	63	40	32	55	45	a b c d
Lifestyle	134	70	71	76	61	48	77	61	b c d
Legal regulations (insurance)	75	39	49	53	17	14	51	41	a c d
Financial problems	17	9	21	23	19	15	48	39	a c d
Meeting possibilities peers	22	11	45	48	29	23	79	64	a b c d

[a] significant different between AYAs (POP vs OCC)
[b] significant different between older adults (POP vs OCC)
[c] significant different between POP-groups (AYAs vs older adults)
[d] significant different between OCC-groups (AYAs vs older adults)

Figure S2. Impact of searching cancer-related information on the internet for AYAs and older adults stratified by POP and OCC groups.

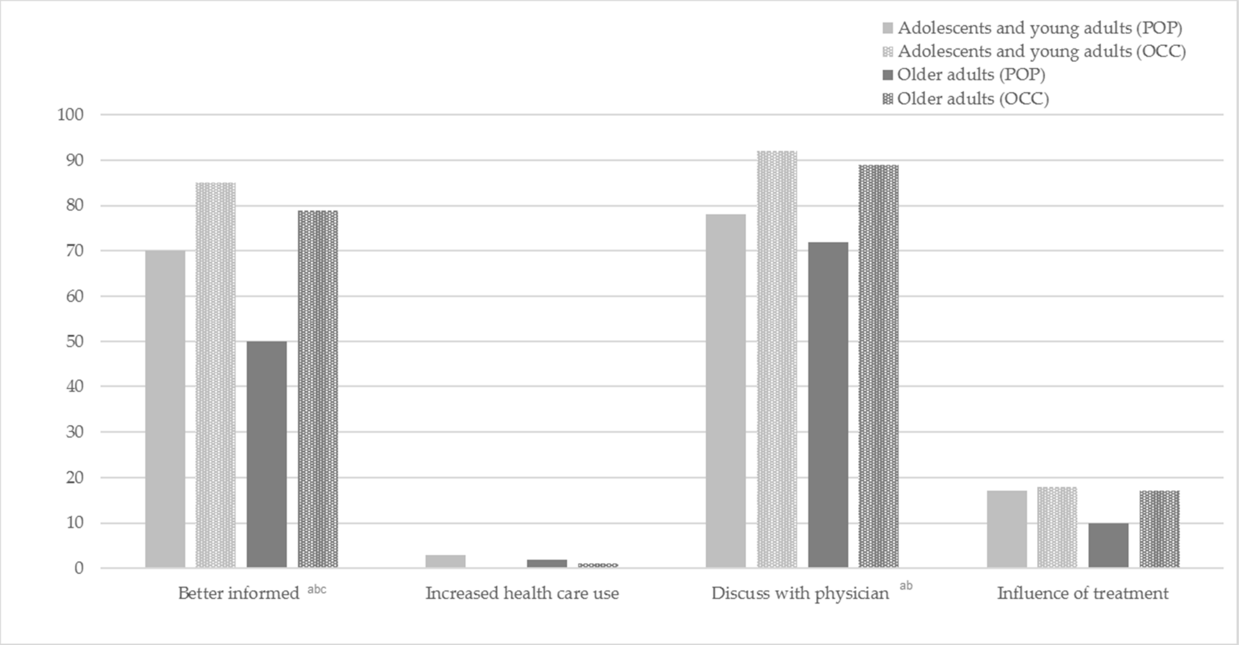

[a] significant different between AYAs (POP vs OCC)

[b] significant different between older adults (POP vs OCC)

[c] significant different between POP-groups (AYAs vs older adults)

[d] significant different between OCC-groups (AYAs vs older adults)

Figure S3. Unmet eHealth needs by AYAs and older adults stratified by POP and OCC groups.

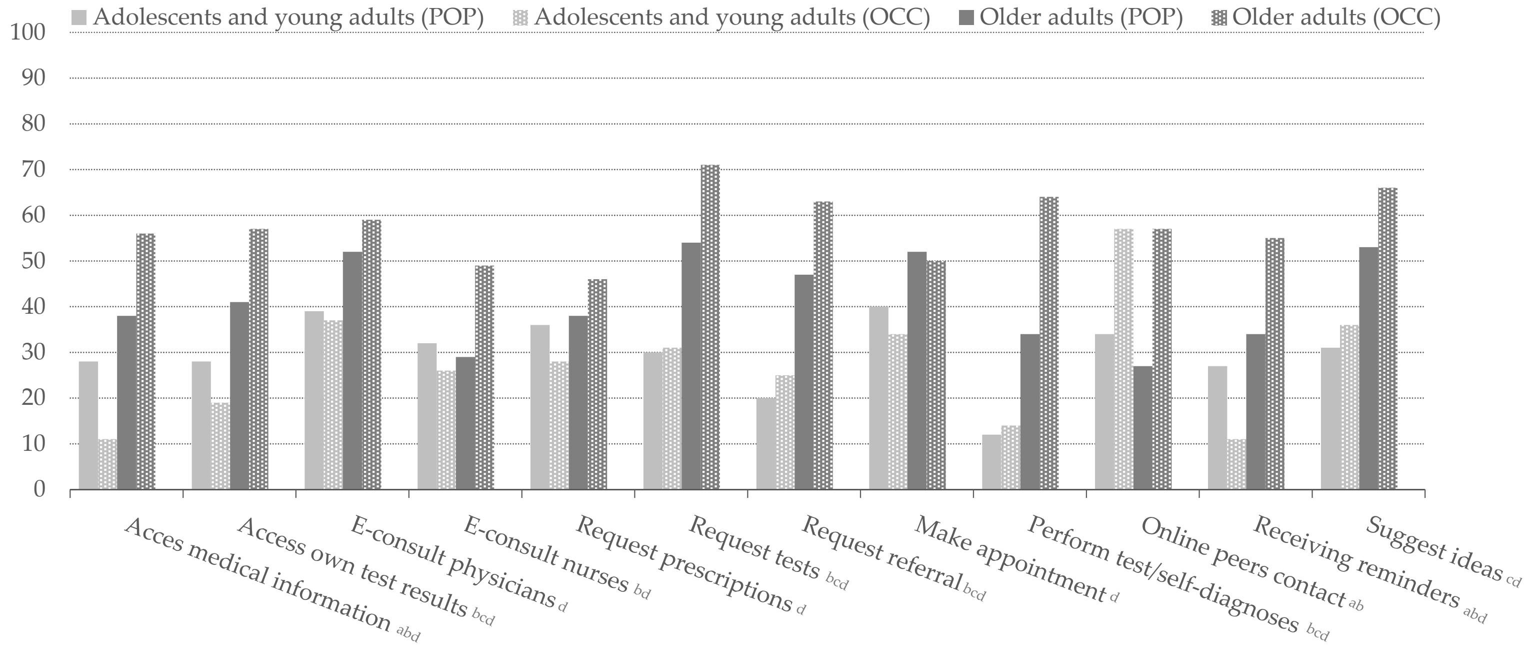

Supplement: Supplementary file 1 [file cancers-13-06308-s001.zip › cancers-1474170-supplementary.pdf]
